# Supplementary material for: Serum Analytes of American Mink (Neovison Vison) Challenged with Aleutian Mink Disease Virus
Source: Animals (Basel). 2022 Oct 11;12(20):2725. doi: 10.3390/ani12202725 (PMC9597810; doi:10.3390/ani12202725)
Supplement: Supplementary file 1 [file animals-12-02725-s001.zip › animals-1901559-supplementary.pdf]

Supplementary Table S1. Reported concentrations of some blood analytes in AMDV-infected and non-infected mink

TP g/l	ALB g/l	ALKP U/l	ALT U/l	BUN mmol/l	CREA µmol/L	CHOL mmol/l	GLU mmol/l	Ca mmol/l	PHOS mmol/l	Sex[†]	Ref.
60.0	27.7	76.0	121.9	11.9	82.2	6.49	4.42	2.24	2.08	M&F	1[§]
62.7	27.6	-	-	-	-	-	-	-	-	F	2[§]
62.4	27.4	91.8	-	9.7	46.2	-	-	-	-	?	3[§]
81.8	17.0	-	-	-	-	-	-	-	-	?	4[§]
57.0	33.0	-	-	-	-	-	-	-	-	?	4
-	-	201.7	145.8	-	-	-	-	-	-	F	5[§]
62.0-70.0	-	-	100-170	6.0-11.0	-	5.5-7.0	8.0-9.5	-	-	F	6
69.2-78.6	-	-	82-153	6.6-8.8[‡]	46.9-68.5	-	7.2-8.2	2.6-2.7	-	F	7
57.0-74.0	-	-	147-353	3.9-7.1[‡]	50.0-59.0	-	-	-	-	M	8
59.4	29.8	-	71.6	5.70	62.76	-	6.99	2.38	1.71	M	9
60.9	30.0	-	80.0	6.08	55.69	-	6.89	2.36	1.67	F	9
-	-	-	13-161	4.2,4.9[‡]	-	-	-	-	-	F	10
68.0-72.0	-	-	70-360	4.6-11.9[‡]	-	-	6.6-9.5	-	-	M	11
69.8-85.3	32-37	152-165	-	-	-	3.98-9.27	-	-	-	M	12
60.4-71.6	-	-	109-214	-	-	-	-	-	-	M&F	13
-	-	-	-	4.0-14.0[‡]	60.0-78.0	-	-	-	-	F	14
53.0-76.0	27-42	-	142-385	3.2-7.6[‡]	38.0-67.0	-	-	-	-	M&F	15
68.1	-	-	133	2.4[‡]	85.0	5.0	8.96	-	-	M	16
66.1	-	-	255	2.8[‡]	71.0	4.8	9.30	-	-	F	16
65.4-85.1	-	67.1	126	9.7	65.9-99.0	-	2.3-7.2	-	1.33	F-L	17
62.3	-	55.8	79.7	5.9	61.5	-	5.8	-	1.30	F	17
61.0	-	-	-	6.9[‡]	69.4	-	5.3	-	5.9	F-L	18
72.4	33.8	-	91.9	7.6[‡]	-	-	5.80	2.45	1.85	F	19
74.2	42.4	-	112.9	8.3[‡]	-	-	9.14	2.70	2.78	M	19
-	-	81-134	698-233	50.5-77.1	-	-	-	-	-	M	20
-	-	-	-	4-17[‡]	58.0-74.0	-	-	-	-	F	21
-	-	-	-	8.2-14.1[‡]	66.9-82.6	-	-	-	-	F-L	22
-	-	-	-	4.9[‡]	64.0	-	-	-	-	F	22
-	-	-	-	6.0-12.2[‡]	108-148.7	-	-	-	-	F-L	23
-	-	-	-	8.3[‡]	110	-	-	-	-	F	23
-	-	-	-	-	-	-	10.1-12.7	-	-	F	24

[§]AMDV-infected

[†]Male (M), female (F) and lactating females (F-L)

[‡]Plasma or blood urea

1-Overall means in the current study

2- Serum of male and female black mink inoculated with AMDV and tested at 218 dpi [70].

3- Serum of black female mink inoculated with AMDV and test at 451 dpi, n=53 [13].

4- Serum proteins in AMDV-infected (n=18) and non-infected (n=18) Aleutian color mink [67]

5- The means were calculated using the reported values for 10 AMDV-infected mink [79].

6- Two-year-old female mink which have been previously selected for high or low residual feed intake (RFI) were fed ad libitum or restrictively at some periods during the 6 weeks of the experiment (16 high and 14 low RFI). Each plasma analyte values are estimates of minimum and maximum values from six graphs [33]

7- Smallest and largest means of serum in three control groups of pregnant mink measured over three years. The activities of ALT were 1.4, 1.6 and 2.6 µKat/L which were converted to 82.4, 94.1 and 152.9 U/l, respectively [123].

8- Scanblack male mink which were fed 5 diets with different protein levels over 3 years and sampled twice each year. Plasma activity of ALT was estimated from graphs (2 to 6 µkat/l) and were converted to U/L (147.6 to 353.0) [59].

9- Serum of brown untamed captive male (n=80) and female mink (n=80) were tested after 12 h of fasting. BUN (15.2 & 16.2), GLU (125.8 & 123.9), Ca (9.54 and 9.47) and PHOS (5.29 and 5.19) were reported as mg/dl and were converted to mmol/l. CREA was converted from mg/dl (0.71 & 0.63) to µmol/l. ALT was reported as µ/l, which was assumed to be U/l [105].
10- Free-living mink were captured and maintained on the same farm (n=7) as captive mink (n=10), fed the same diet for one month before sampling [90].
11- Plasma analytes of male mink which were fed high or low protein diets and tested 5 times between July and December. Blood protein was measured only in December. ALAT values were estimated from a graph [44].
12-Serum analytes in healthy dark male mink supplemented with seven levels of copper [99].
13- Serum TP of male and female mink (n=194) of standard genotype which were fed one of the seven diets. ALT was measured only in males [37].
14- Adult females mink fed high or low protein diets, raw or cooked, and plasma concentration of CREA and urea were determined from graphs [39].
15- Plasma analytes of male and female mink fed four diets with different protein contents and measured in July, September, October and December [50].
16- In plasma of a control group of black mink before the start of a feeding experiment [38].
17- Healthy control female mink sampled on weeks 4, 5 and 6 of lactation, and terminal values of non-lactating and lactating mink [71].
18-Analytes in plasma of healthy lactating mink (n=17)[78].
19- Serum analytes in 55 female and 30 male standard dark 7-month old mink [46].
20- The smallest and largest means of analytes measured in plasma of male mink on three farms fed diets with different bacterial counts. ALKP, ALT and GGT were reported as Ukat/l and were converted to U/l [49].
21- Adult female pastel mink (n=10) were fed high or low protein diets, followed by fasting during which blood samples were collected six times, from 2 to 48 h of fasting, and plasma concentrations of urea and CREA were measured. The values were estimated from graphs [45].
22-Plasma concentrations of urea and CREA were measured on days 26, 34, 41 and 44 postpartum, and in barren females fed normal levels of salt [73].
23- Plasma concentrations of CREA and urea of lactating dams (n=30) on days 35, 42, 49 and 56 post-partum and barren females (n=10) which were sampled corresponding to day 35 of the lactating dams [72].
24- Plasma glucose in the control group (ad lib feeding) of 10 female mink measured over six weeks [98].
